# Ocular Dirofilariasis after Clinically Manifested Subcutaneous Migration of the Parasite: A Case Report

**Published:** 2020

**Authors:** František ONDRISKA, Vojtech BOLDIŠ, Marta STANISLAVOVÁ, Daniela ANTOLOVÁ, Martina MITERPÁKOVÁ, Anton HANÁČEK, Soňa VEŠPEROVÁ, Ivan JANČOVIČ

**Affiliations:** 1. Department of Laboratory Testing Methods in Health Care, Faculty of Health Care and Social Work, Trnava University, Trnava, Slovakia; 2. Department of Parasitology, Medirex (Ltd), Bratislava, Slovakia; 3. Department of Ophthalmology, University Hospital of Bratislava, Bratislava, Slovakia; 4. Department of Zoonotic Diseases, Institute of Parasitology SAS, Košice, Slovakia; 5. Department of Ophthalmology, Faculty Hospital of Trnava, Trnava, Slovakia; 6. Department of Infectology, Faculty Hospital of Trnava, Trnava, Slovakia; 7. ENT Outpatient Department, Bánovce nad Bebravou, Slovakia

**Keywords:** Dirofilariasis, *Dirofilaria repens*, Subcutaneous migration, Ocular infection, Slovakia

## Abstract

*Dirofilaria repens* is the causative agent of human subcutaneous or, less often, ocular dirofilariasis. The work presents a rare case of ocular dirofilariasis manifested by previous subcutaneous migration accompanied by severe headache symptoms. In February 2017, a 58-yr-old man from Trnava region, western Slovakia, noticed red and itchy stripes on his left leg. Inflamed but painless stripes disappeared and showed up again every 5–7 days, migrating gradually towards the head. Approximately one month after the first skin′s alterations, strong pain in the left temple, with the swelling of the left face and the enlargement of mandibular lymph nodes appeared. Several days later, the patient felt excruciating pain of the right eyeball accompanied by strong nausea and subsequent vomiting. Ocular examination revealed the presence of a live worm in the subconjunctival space and morphological and molecular analyses of extracted helminth confirmed *D. repens* as etiological agent of the infection. According to clinical manifestation of the infection, it could be supposed that ocular form of the disease was the result of the migration of a parasite through the subcutaneous tissues. Moreover, a rare phenomenon of lymphadenitis of underlying lymph nodes and the swelling of left face accompanied the migration.

## Introduction

A number of different helminthic species can affect human eye. Some of them, like *Wuchereria bancrofti*, *Brugia malayi*, *Loa loa*, and zoonotic *Onchocerca volvulus* and *Angiostrongylus cantonensis,* represent public health problem in developing countries; the spread of other, e.g. *Thelazia callipaeda* or *Dirofilaria repens*, are connected with climate and environmental changes (1).

Dirofilariasis is considered fast spreading disease with the causative agent being transmitted by mosquitoes of various species. The most frequently detected *Dirofilaria-*species parasites in humans is *D. repens*, which causes subcutaneous and ocular form of the infection (2). Since 2007, when the first autochthonous case of human dirofilariasis in Slovakia was recorded, a total of 16 19 human cases caused by *D. repens* have been reported in Slovakia; four of them of ocular form (3,4; Antolová, Miterpáková and Ondriska – personal communication). Concerning ocular dirofilariasis, adult parasites are often present subconjunctivally, intravitreally or directly in orbit (5). Presence of the parasite in eye structures is clinically manifested mainly by itching and inflammatory reaction, which may result in impaired vision of the patient.

Dirofilarial infections are relatively often accompanied with the parasite subcutaneous migration, and even cutaneous larva migrans syndrome was recorded in Slovak patient recently (6). Herein we present a rare and remarkable case of ocular dirofilariasis manifested by foregoing subcutaneous migration accompanied by severe headache symptoms.

## Case presentation

In February 2017, a 58-yr-old man from Trnava region, western Slovakia, noticed red and itchy stripes appeared on his left leg, 10 cm below the crotch. Within next five days, the stripes disappeared and showed up again, first on the right side of the back and later under the scapula. The lesions were itchy, elon-gated, ascended about 0.5 mm above skin′s surface and reached about 2 cm in length. The nodules under the scapula were not pruritic and reminded of purulent ulcer. Approximately one month after the first skin alterations had been observed; strong pain in the left temple, swelling of the left half of the face and the enlargement of the mandibular lymph nodes appeared. Several days later, the patient had felt excruciating, one-hour lasting pain of the right eyeball, which ceased and reappeared next day. The pain was accompanied by strong nausea and subsequent vomiting.

**Fig. 1:**
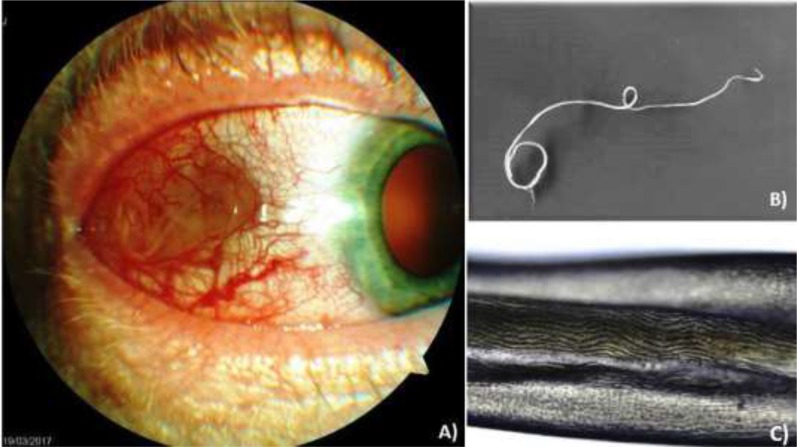
*Dirofilaria repens* worm found in ocular structures of the patient. A) The patient′s eye with a live worm in the subconjunctival space; B) The worm extracted from the right eye; C) Multi-layered cuticle with external longitudinal ridges typical for *D. repens* observed by the microscopic examination

Clinical examination at the Clinic of Ophthalmology revealed the presence of a live worm in the subconjunctival space (Fig. 1A). The patient agreed with all examinations and signed the informed content. No identifying data are presented in the paper. The study and subsequent publication of results was approved by the Ethics Committee of Institute of Parasitology of SAS (No. EK 04/2015).

From the time of the first dermal symptoms to the appearance of the worm in the eye it took about six weeks. Common laboratory findings as well as the numbers of erythrocytes and leucocytes were within the normal range, while the absolute count of eosinophils was slightly increased to 0.69 × 10^9/L (reference value 0.0 – 0.50 × 10^9/L). The extracted helminth was whitish, thin and measured about 8.3 cm in length (Fig. 1B). Based on microscopic examination, the presence of multi-layered cuticle with external longitudinal ridges was observed (Fig. 1C). Preliminary diagnosis of dirofilariasis caused by *D. repens* species was pronounced.

Subsequently, the nematode was sent for molecular analysis. Genomic DNA was isolated by using DNeasy Blood and Tissue Kit (Quiagen, Hilden Germany) according to the manufacturer′s instructions. PCR reactions were performed using 5× FIREPol® Master Mix Ready to Load (SOLIS Biodyne, Estonia). Mitochondrial gene for cytochrome oxidase subunit 1 (*cox1*) was amplified using specific primers for both, *D. immitis* (DI COI-F1: AGT GTA GAG GGT CAG CCT GAG TTA and DI COI-R1: ACA GGC ACT GAC AAT ACC AAT) and *D. repens* (DR COI-F1: AGT GTT GAT GGT CAA CCT GAA TTA and DR COI-R1: GCC AAA ACA GGA ACA GAT AAA ACT) (7). As positive control, DNA isolated from *D. immitis* microfilariae from dog′s blood and DNA from *D. repens* worm isolated from human subcutaneous tissue was used. Amplification consisted of denaturing step (94 °C/2 min), 35 cycles at the temperatures 94°C/30 sec, 57°C/30 sec and 72°C/30 sec and final extension (72 °C/7 min). The presence of approximately 200 bp long band on 1.5 % agarose gel confirmed that our nematode belongs to *D. repens* species.

The patient was treated with antibiotic eye drops (ofloxacin), ophthalmic corticosteroids (dexamethasone), and ophthalmic gel containing dexpanthenol. No new symptoms have been observed after the worm extraction.

From epidemiological point of view, the autochthonous origin of the infection cannot be unambiguously confirmed, because in August 2016 patient had spent his holiday in Calabria, southern Italy.

## Discussion

Until recently, human dirofilariasis was considered rare infection in the territory of Central Europe. Nevertheless, the incidence of the disease is increasing, especially in countries regarded as non-endemic (2, 8). Until in the dogs *D. repens* is mostly located free in subcutaneous tissues, the parasite in human body usually forms a nodule, with the exception of ocular form of the infection.

In case of ocular dirofilariasis, the nematode is considered migratory and not trapped by the host′s defense mechanisms (2). In the eye, *Dirofilaria* worms can be present in eyelid, conjunctiva, inside the bulb, but most often subconjunctivally, with the presence of helminths usually being accompanied by redness, burning, eye pain, itching and sensation of the foreign item in the eye (9,10). The helminths occur in the form of a cystic lesion or are directly visible (3,11), with one, exceptionally two individuals detected (12). Despite of ocular dirofilariasis represents still less common form of the infection in comparison with subcutaneous form, during the last two decades progressive increase of its incidence has been reported in various European countries including Serbia, Greece, Belgium or Denmark (6, 11, 13, 14).

In Slovakia, the first case diagnosed as ocular dirofilariasis was published in 1992 when mobile worm, measured about 9 mm in its length, was removed from vitreous body, even though some doubts were revealed based on the retrospective view of this case (4). Since then, between 2012 and 2017, four other patients with ocular dirofilarial infection were recorded in Slovakia (3, 4). With the exception of the last, here described case, in all of the patients autochthonous origin of the infection was confirmed. Moreover, three of four infected persons lived in southwestern part of the country characterised by warm and humid climate suitable for mosquito-vectors and the parasite development (15). In addition, the majority of infected dogs, with the mean regional prevalence of about 25 %, were found in region of southern Slovakia (16). Seeing that the patient presented in our study also lives in this locality, but stayed also in endemic area in southern Italy, it is not possible to confirm an origin of the infection.

In the event of ocular dirofilariasis, majority of cases are related to subconjunctival localization of the parasite, which is a result of its migration through the human body. Active migration of *D. repens* is phenomenon observed by several authors. For instance, Ermakova et al. (17) in her study describes parasite′s migration in more than 43 % of 266 patients with subcutaneous form of the infection. In more than 17% of the cases, the worms moved for quite long distances with the most common final localization in upper half of the body (head, neck and periorbital area). An interesting case was described also in Slovakia when clinical case of *D. repens* infection was connected with cutaneous larva migrans syndrome accompanied by severe pain, burning and erythema of the skin (6). In our patient, a parasite′s migration was visible in a form of ascended subcutaneous strips. The lesions were approximately 2 cm long and protruded 0.5 mm above the skin surface. The strips appeared every 5–7 days on the back and migrated gradually towards the head and then to the eye. Nodules were inflamed but painless.

A rare phenomenon was the acute headache and the lymphadenitis of the underlying nodes with swelling of the left side of the face. Eye pain is not exceptional in ocular dirofilariasis, but the urgent pain that forced the patient to vomit is not often described. We did not encounter any similar symptoms in the other patients with ocular dirofilariasis in Slovakia, when only asymptomatic migration resulted to the parasite′s appearance in ocular structures connected with eye itching and redness (3,4). However, the parasite pathway to the eye is not yet sufficiently explained. It is assumed, that microfilariae migrate into vitreous body and eye anterior chamber via blood stream, where they develop to the adult helminths, or the presence of the worm in the eye structures is a result of the migration of an adult stage of the parasite through the subcutaneous tissues (13). According to the clinical manifestation observed in here reported patient, it could be supposed the eye infection developed after the migration of the adult worm.

Concerning differential diagnosis of eye-worm infections, the causative agent can be definitively determined only after surgical extraction of the host tissues. Subsequent microscopic observation and histological examination can easily distinguish *D. repens* from other helminths based on characteristic morphologic features (18). Nevertheless, in cases when extirpated biological material is damaged or incomplete, DNA analysis by molecular methods is inevitable for definite diagnosis.

## Conclusion

Herein presented case confirmed that ocular *D. repens* infection can be the result of the migration of an adult parasite through the subcutaneous tissues. Moreover, rare symptoms of the lymphadenitis of underlying lymph nodes and the swelling of the face are reported.
